# Effects of red–blue light ratio on seedling growth and specialized metabolism in tissue-cultured Nauclea officinalis

**DOI:** 10.3389/fpls.2026.1818221

**Published:** 2026-04-22

**Authors:** Hanming Zhang, Tianqi Huo, Wenjie Yu, Weili Yang

**Affiliations:** 1 Key Laboratory of Tropical Translational Medicine of Ministry of Education, Hainan Provincial Key Laboratory of R&D of Tropical Herbs, School of Pharmaceutical Sciences, Hainan Medical University, Haikou, China; 2 Tropical Environment and Health Laboratory, School of Public Health, Hainan Medical University, Haikou, China

**Keywords:** light quality, *Nauclea officinalis*, secondary metabolism, strictosamide, transcriptome

## Abstract

Light quality plays an important role in plant growth and metabolism, yet its integrated effects on physiology and molecular regulation in *Nauclea officinalis* remain unclear. In this study, tissue-cultured seedlings were exposed to white (W), blue (B), red (R), and mixed red–blue light treatments (R1:B1, R1:B2, and R2:B1). The results showed that blue light and the R1:B2 treatment significantly upregulated the light-harvesting genes *LHCB5* and *CAB7*, as well as the chlorophyll biosynthesis-related gene *HEMA1*. Photosynthetic pigment accumulation showed a clear temporal response, peaking at 20 d; blue light increased chlorophyll a, chlorophyll b, and total chlorophyll by 49.81%, 150.16%, and 80.48%, respectively, compared with the control, though these effects weakened by 30 d. Total flavonoid and total phenolic acid contents both increased with time, but they displayed distinct response patterns to light quality: At 30 d, total flavonoid accumulation was highest under R1:B2, while total phenolic acid content remained relatively high under W, R, B, and R2:B1 but was lower under R1:B1 and R1:B2. Transcriptomic analysis showed that core flavonoid biosynthetic genes, (including *CHS*, *CHI*, *FLS/F3H*, *F3’H*, *DFR*, *ANS*, *LDOX*, and ANR), exhibited clear treatment-specific expression patterns, and several genes showed relatively higher expression under R1:B2 and R2:B1, consistent with the flavonoid accumulation trend. Red light mainly suppressed gene expression, whereas mixed red–blue treatments promoted genes associated with photosynthesis and secondary metabolism. Seedling growth was also most strongly promoted under R1:B2, with significant increases in biomass, plant height, and root length (*P* < 0.05). Overall, the R1:B2 red-blue ratio was the most favorable for coordinating growth, photosynthetic performance, and secondary metabolite accumulation in tissue-cultured *N. officinalis* seedlings under controlled conditions, providing a reference for seedling-stage light regulation during controlled cultivation.

## Introduction

Plants use light not only as an energy source but also as a developmental signal that orchestrates growth and metabolism. The advent of adjustable light-emitting diode (LED) lamps has revealed that red and blue wavelengths exert distinct yet often complementary effects. Red light promotes stem elongation and carbohydrate accumulation, whereas blue light enhances photosynthetic efficiency and can induce stress-responsive antioxidant systems under certain conditions (Bottiglione et al., 2023; Rahman et al., 2025). Blue irradiation activates cryptochromes and phototropins, leading to HY5 (ELONGATED HYPOCOTYL 5)/MYB (v-myb avian myeloblastosis viral oncogene homolog) -mediated induction of phenylpropanoid-biosynthetic genes such as *PAL* (phenylalanine ammonia-lyase), *CHS* (chalcone synthase), *4CL* (4-coumarate-CoA ligase), while red light activates phytochromes, stabilises HY5, and stimulates terpenoid biosynthesis via the methyl-erythritol phosphate pathway. These molecular effects translate into agronomic outcomes: mixed red-blue spectra often outperform monochromatic treatments because red and blue signals act synergistically. This synergy arises from the co-action of phytochrome B and cryptochrome photoreceptors (Chenge-Espinosa et al., 2018); a brief blue-light pulse on red-grown seedlings can elicit persistent transcriptomic and growth changes via HY5, HYH (HY5 HOMOLOG), and SPA (SUPPRESSOR OF PHYTOCHROME A-105) regulators (Sellaro et al., 2009). In practice, cucumber seedlings under red-blue combinations display reduced height but increased stem diameter and root dry weight compared with red or blue alone, and mixed spectra optimise photosynthesis and biomass at approximately 90% red and 10% blue (Wang et al., 2024). Poplar seedlings exposed to red or red-dominant light contain the highest soluble sugar and sucrose concentrations, whereas blue-dominant mixes lower the light-compensation point and enhance photosynthetic rates (Xu et al., 2025). Blue-enriched spectra can also stimulate flavonoid accumulation in *Scutellaria baicalensis* (Zhang et al., 2022), whereas red light promotes amino acid synthesis and phenylpropanoid gene expression in lettuce and chicory callus (Hernández et al., 2024; Abdelhamid et al., 2025). Optimal ratios vary; for the medicinal species *Glehnia littoralis*, a red:blue ratio of 7:5 maximised shoot fresh weight, leaf area, and coumarin accumulation (Yeom and Oh, 2025).

These diverse responses underscore the need to tailor LED spectra to specific crops. In leafy vegetables such as pak choi, mixed red-blue LEDs maximise glucosinolate, phenolic, and anthocyanin contents and provide the highest antioxidant activity (Bungala et al., 2024). For medicinal plants, light quality is especially critical because secondary metabolites determine therapeutic value. Studies on *Glycyrrhiza* and basil microgreens show that red-dominant mixes (4R:1B) enhance plant height and biomass, whereas blue light suppresses growth (Jiang et al., 2025) but can increase antioxidant capacity and phenolic content (Thongtip et al., 2024). Conversely, elevated blue light accelerates wheat spike development and up-regulates genes involved in chlorophyll and anthocyanin synthesis (Sun et al., 2024). Despite this growing body of evidence, research on woody medicinal species lags behind. *Nauclea officinalis Pierre*, a Rubiaceae tree native to southern China, is used to treat fever, pneumonia, and diarrhoea and contains indole alkaloids such as strictosamide and vincosamide, pentacyclic triterpenoids, and phenolic acids with anti-inflammatory, antiviral, and antimalarial activities (Liu et al., 2022). Among these metabolites, strictosamide is a medicinally relevant compound closely associated with monoterpene indole alkaloid (MIA)-related metabolism, in which tryptamine-derived and terpenoid-derived precursors are condensed and subsequently modified through stepwise enzymatic reactions. Rising demand has spurred interest in controlled cultivation. In the context of good agricultural practice (GAP) -based production of medicinal materials, the consistency of raw medicinal materials is closely related to standardized management from the source. For woody medicinal species, the seedling stage is a key phase influencing later stand uniformity and quality formation. Therefore, spectral regulation at the nursery stage may serve as a controllable approach to improving seedling consistency and to supporting more stable raw material production in subsequent cultivation yet the effects of spectral composition on its growth, pigment accumulation, active metabolite accumulation, and underlying biosynthetic pathways remain unclear. Despite increasing interest in LED-based spectral regulation, evidence remains limited for woody medicinal species, particularly regarding the coordination between seedling growth and active metabolite biosynthesis. In this study, we used tissue-cultured seedlings of *N. officinalis* to evaluate how monochromatic and mixed red-blue spectra affect growth traits, photosynthetic characteristics, total flavonoids, total phenolic acids, and strictosamide accumulation under controlled conditions. By integrating physiological measurements with transcriptomic and quantitative real-time PCR (qRT-PCR) analyses, we aimed to clarify the seedling-stage response pattern of *N. officinalis* to light quality and to identify a suitable spectral combination for controlled cultivation studies. Rather than establishing a universally applicable lighting strategy, this study focuses on the seedling-stage response pattern of a woody medicinal species under controlled conditions. Its value lies in integrating physiological traits, active metabolite accumulation, and related transcriptional responses to provide a species-specific reference for *N. officinalis*.

## Materials and methods

### Experimental materials

Plant Materials and Culture Conditions: Seeds of *N. officinalis* were surface-sterilized by rinsing with sterile water (2–3 times), immersing in 75% ethanol for 30 s, followed by 8% NaClO for 15 min, and finally rinsing 10–15 times with sterile water. The sterilized seeds were inoculated onto Murashige and Skoog (MS) medium supplemented with 0.1 mg/L 1-naphthaleneacetic acid (NAA). Upon germination, seedlings were subcultured on woody plant medium (WPM) containing 0.1 mg/L NAA and 0.1 mg/L indole-3-butyric acid (IBA). After the root systems were well-developed, plantlets were transferred to WPM medium supplemented with 0.1 mg/L IBA for subsequent experiments. The culture environment was maintained at 24 ± 2 °C with a relative humidity of 75 ± 5%.

Uniform tissue-cultured seedlings with well-developed roots and similar size were randomly assigned to six light treatments: white light (W), monochromatic blue light (B), monochromatic red light (R), and three combined red–blue light treatments with photon flux ratios of 1:1 (red1:blue1,R1:B1), 1:2 (red1:blue2,R1:B2), and 2:1 (red2:blue1,R2:B1). Combined treatments were generated by simultaneous illumination with red and blue LEDs, and the ratios were defined on the basis of photosynthetic photon flux density (PPFD). The blue LED had a spectral range of 450–470 nm with a peak at 450 nm, whereas the red LED had a spectral range of 620–660 nm with a peak at 660 nm. White light was provided by a broad-spectrum white LED source covering 400–700 nm, with a dominant blue peak around 450 nm and a broad emission band between 500 and 650 nm. For all treatments, the total PPFD was maintained at 60 μmol m^−^² s^−^¹ under a 12 h light/12 h dark photoperiod. Each treatment included three biological replicates, with 15 bottles of tissue-cultured *N. officinalis* seedlings per replicate.

### Transcriptome sequencing and analysis

Total ribonucleic acid (RNA) was isolated from seedlings harvested at 30 d using TRIzol reagent, and RNA quality was assessed with an Agilent 2100 Bioanalyzer. Only samples with an RNA integrity number (RIN) > 7.0 and total RNA amount > 1 μg were used for subsequent analysis. Strand-specific cDNA libraries were constructed by enriching poly(A) mRNA, followed by fragmentation and dUTP-based second-strand synthesis. The libraries (300 ± 50 bp) were amplified and sequenced on an Illumina NovaSeq 6000 platform with 150-bp paired-end reads following the manufacturer’s protocols. Clean data were processed to identify differentially expressed genes (DEGs) for subsequent bioinformatics analysis.

### qRT-PCR validation

To validate the reliability of the transcriptome sequencing (RNA-Seq) data, thirteen DEGs were selected for qRT-PCR verification. These selected genes included light-harvesting complex II protein 5 (*LHCB5*), chlorophyll a/b-binding protein 7 (*CAB7*), photosystem I reaction center protein N (*PSAN*), 5-aminolevulinate synthase (*HEMA1*), cytochrome P450 (*CYP*), strictosidine synthase (*STR*), tryptophan decarboxylase (*TDC*), secologanin synthase (*SLS*), short-chain dehydrogenase/reductase (*SDR*), Arabidopsis thaliana gene locus *At5g45910* (*At5g45910*), ethylene-responsive transcription factor (*ELI5*), 4-coumarate-CoA ligase-related gene (*ICMEL2*), and ferredoxin 3 (*FDX3*).

The ubiquitin 8 (*UBQ8*) gene was used as the internal reference gene for data normalization, which effectively eliminated the interference caused by variations in RNA extraction efficiency and reverse transcription levels, ensuring the accuracy and reproducibility of gene expression quantification.

All specific primers for the selected DEGs and the internal reference gene were designed and synthesized by Accurate Biology (China) (**Supplementary Table S1**).

### Determination of growth indicators

At 30 d, the plant height, root length, leaf length, leaf width, and leaf number of *N. officinalis* seedlings were measured using a vernier caliper. Fresh weight was measured by an electronic balance to determine the mass of each part of the plant. The method for measuring the dry weight of plants was to place the plants in an electric constant temperature drying oven, set the temperature to 80 °C, and dry for 48 hours until the constant weight was reached. At the same time, the moisture content is calculated (**Supplementary Table S1**).

### Determination of photosynthetic indicators

At 10, 20, and 30 days after treatment, Soil Plant Analysis Development (SPAD) values and chlorophyll content were determined in *N. officinalis* leaves. SPAD values were measured using a TYS-B chlorophyll analyzer. For each seedling, the third fully expanded leaf from the apex was selected, and measurements were taken at the widest part of the leaf blade while avoiding the midrib.

Each treatment comprised three biological replicates. Chlorophyll content was determined via ethanol extraction: 0.5 g fresh leaf tissue (collected at 10, 20, and 30 d) was immersed in 10 mL absolute ethanol, incubated in darkness for 24 h until full decolorization, and the extract absorbance measured at 663 nm and 645 nm. Each treatment was analyzed in triplicate.

### Determination of total phenolic acids and total flavonoids

At 10, 20, and 30 days post-treatment, leaves were harvested, dried, and ground into powder. Dried leaf powder (1.0 g) was subjected to three times with 20 mL of 60% ethanol for 60 min each time. The supernatants were combined, centrifuged, and diluted 10-fold with 60% ethanol. A 2 mL aliquot of the diluted extract was mixed with 1 mL of Folin–Ciocalteu reagent and incubated in the dark for 10 min. Then, 0.8 mL of Na_2_CO_3_ solution was added, and the mixture was brought to a final volume of 10 mL with distilled water. After incubation in the dark for 45 min, the absorbance was measured at 765 nm. Total phenolic content was calculated using a gallic acid standard curve.

Total flavonoid content: At 10, 20, and 30 days post-treatment, leaves were harvested, dried, and ground into powder. Leaf powder (1.0 g) was ultrasonically extracted with 10 mL of 95% ethanol for 30 min, and the filtrate was diluted 10-fold with 95% ethanol. For the assay, 2 mL of the diluted extract was mixed with 0.5 mL of 5% NaNO_2_ and allowed to react for 6 min, followed by the addition of 0.3 mL of 10% Al(NO_3_)_3_ for another 6 min, and then 4 mL of 4% NaOH for 20 min. The absorbance was measured at 510 nm. Total flavonoid content was determined using a rutin standard curve.

### Strictosamide determination

Leaves of *N. officinalis* harvested at 30 days after treatment were oven-dried and ground into powder. The powder was extracted with 65% ethanol at a material-to-solvent ratio of 1:100 (g/mL) by ultrasonic extraction for 40 min. After filtration, the extract volume was adjusted to the original volume to compensate for solvent loss. Subsequently, 1 mL of the extract was diluted to 10 mL with the mobile phase (acetonitrile:0.1% phosphoric acid = 25:75, v/v), filtered through a 0.22 μm membrane, and stored at 4 °C before high-performance liquid chromatography (HPLC) analysis. The strictosamide standard stock solution (160 μg/mL) was prepared by dissolving 1.6 mg of strictosamide reference standard in the same mobile phase.

HPLC Conditions Chromatographic analysis was performed using a Phenomenex Luna C18(2) column (150 mm × 4.6 mm, 5 μm). The mobile phase consisted of acetonitrile and 0.1% aqueous phosphoric acid (25:75, v/v) with isocratic elution. The flow rate was maintained at 1.0 mL/min, and the column temperature was set to 25 °C. The detection wavelength was 226 nm, and the injection volume was 20 μL.

### Statistical analysis

All experiments were performed with three biological replicates. Data are expressed as the mean ± standard deviation (SD). Microsoft Excel 2016 was used for data compilation, and GraphPad Prism 8.0 was employed for statistical analysis and figure generation.

## Results

### Differential expression of different light quality transcriptomes

**Figure 1:**
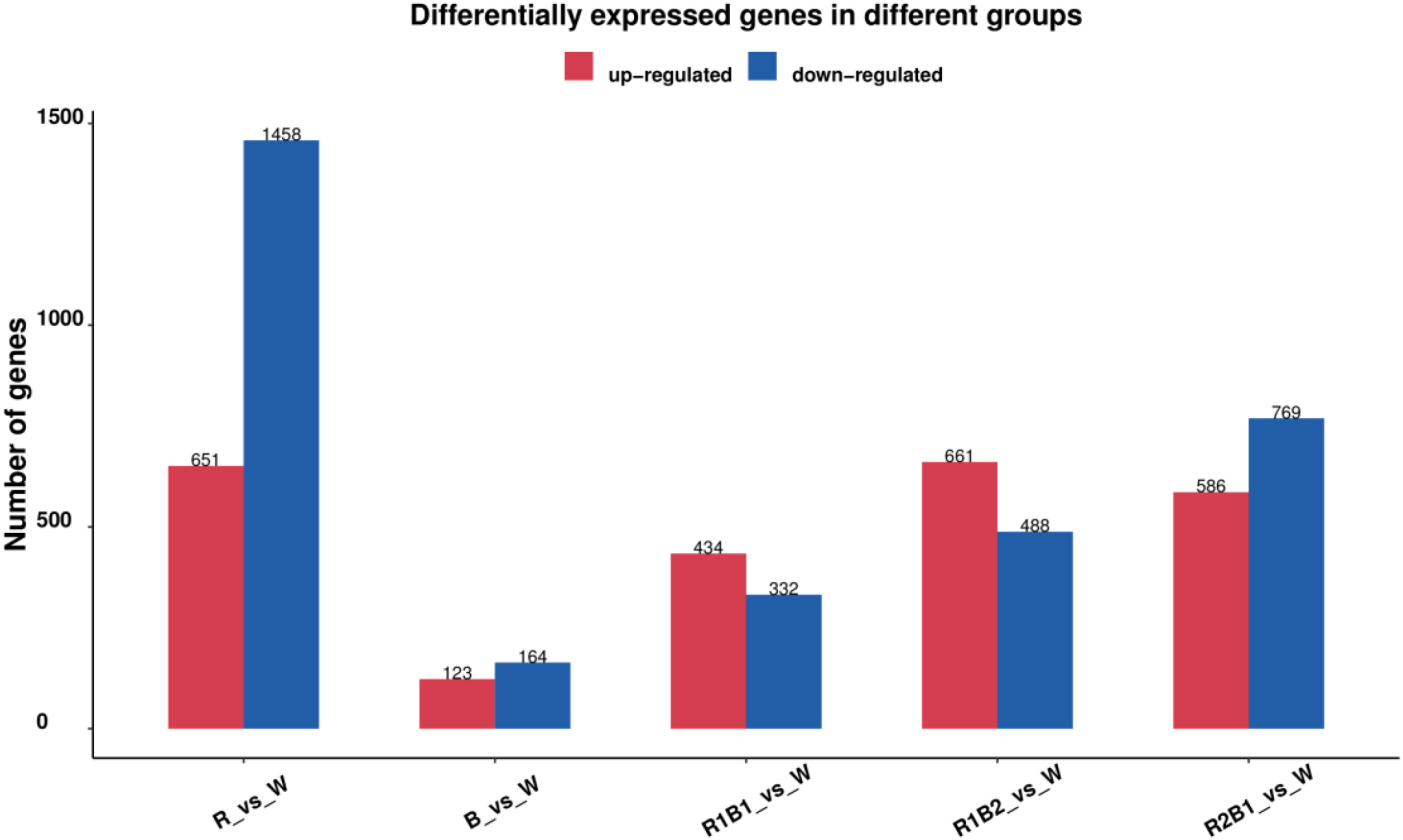
Transcriptomic analysis of differentially expressed genes (DEGs) in *N. officinalis* under different light qualities. The number of DEGs in different comparison groups is shown; the red and blue bars represent upregulated and downregulated genes, respectively.

We identified DEGs in *N. officinalis* using |log_2_FC| > 1 and *P* < 0.05; we visualized them with bar and volcano plots (**Figure 1**). R compared to W showed strong down-regulation (1,458 down vs 651 up), whereas B compared to W produced the fewest DEGs (123 up, 164 down). In mixed spectra, up-regulation dominated in R1:B1 compared to W (434 up, 332 down), R1:B2 compared to W (661 up, 488 down), and R2:B1 compared to W (586 up, 769 down), consistent with transcript suppression under red light and activation under combined red–blue light.

### KEGG enrichment under different light qualities

**Figure 2:**
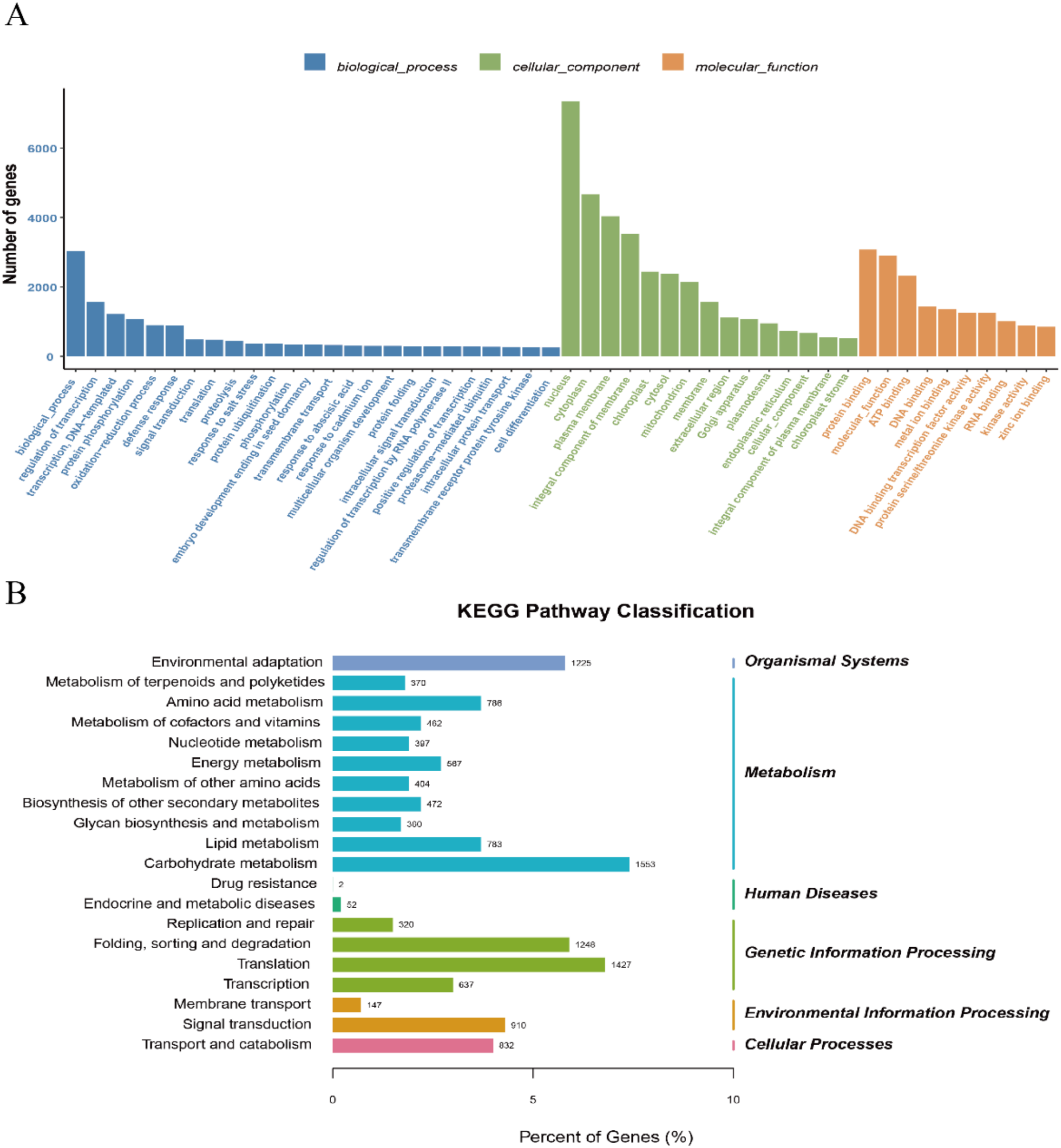
Functional enrichment analysis of DEGs in *N. officinalis* under different light qualities. **(A)** Gene Ontology (GO) classification of DEGs; the x-axis indicates GO terms, grouped into biological process (blue), cellular component (green), and molecular function (orange). **(B)** KEGG pathway enrichment analysis of DEGs, presented as –log10(*P* -value).

To characterize pathway-level regulation under different red–blue ratios, we performed Kyoto Encyclopedia of Genes and Genomes (KEGG) enrichment analysis on DEGs from each treatment (**Figure 2B**). Under blue light, DEGs were mainly enriched in carotenoid biosynthesis, phenylpropanoid biosynthesis, photosynthesis, and circadian rhythm. Under red light, enrichment was centered on starch and sucrose metabolism, nitrogen metabolism, and plant hormone signal transduction. Mixed spectra produced broader pathway signatures: R1:B1 enriched sesquiterpenoid and triterpenoid biosynthesis, chlorophyll metabolism, glyoxylate and dicarboxylate metabolism, and linoleic acid metabolism; R1:B2 enriched photosynthesis, porphyrin and chlorophyll metabolism, and photosynthesis-antenna proteins; and R2:B1 enriched circadian rhythm–plant, starch and sucrose metabolism, and phenylpropanoid biosynthesis, supporting treatment-specific coordination between photosynthetic/light-adaptive and metabolic/hormonal pathways.

### GO enrichment under different light qualities

To functionally categorize DEGs, we conducted Gene Ontology (GO) enrichment analysis and visualized the results as bar plots (**Figure 2A**). Enriched biological-process terms were dominated by regulation of transcription (DNA-templated), protein phosphorylation, and oxidation–reduction processes. Enriched cellular-component terms primarily mapped to the nucleus, cytoplasm, plasma membrane, and integral components of membrane. Enriched molecular-function terms were mainly protein binding, ATP binding, and DNA binding, indicating that the DEG set is largely associated with transcriptional control, signaling-related phosphorylation, and core binding functions across major cellular compartments. Overall, these transcriptomic results indicate that monochromatic red light mainly exerted a suppressive effect on gene expression, whereas mixed red–blue spectra, especially R1:B2, activated pathways related to photosynthesis, chlorophyll metabolism, and specialized metabolism. This pattern suggests that combined spectral signals better support coordinated physiological regulation in *N. officinalis* than single-wavelength red light.

### Growth impact of light quality on *Nauclea officinalis*

**Figure 3:**
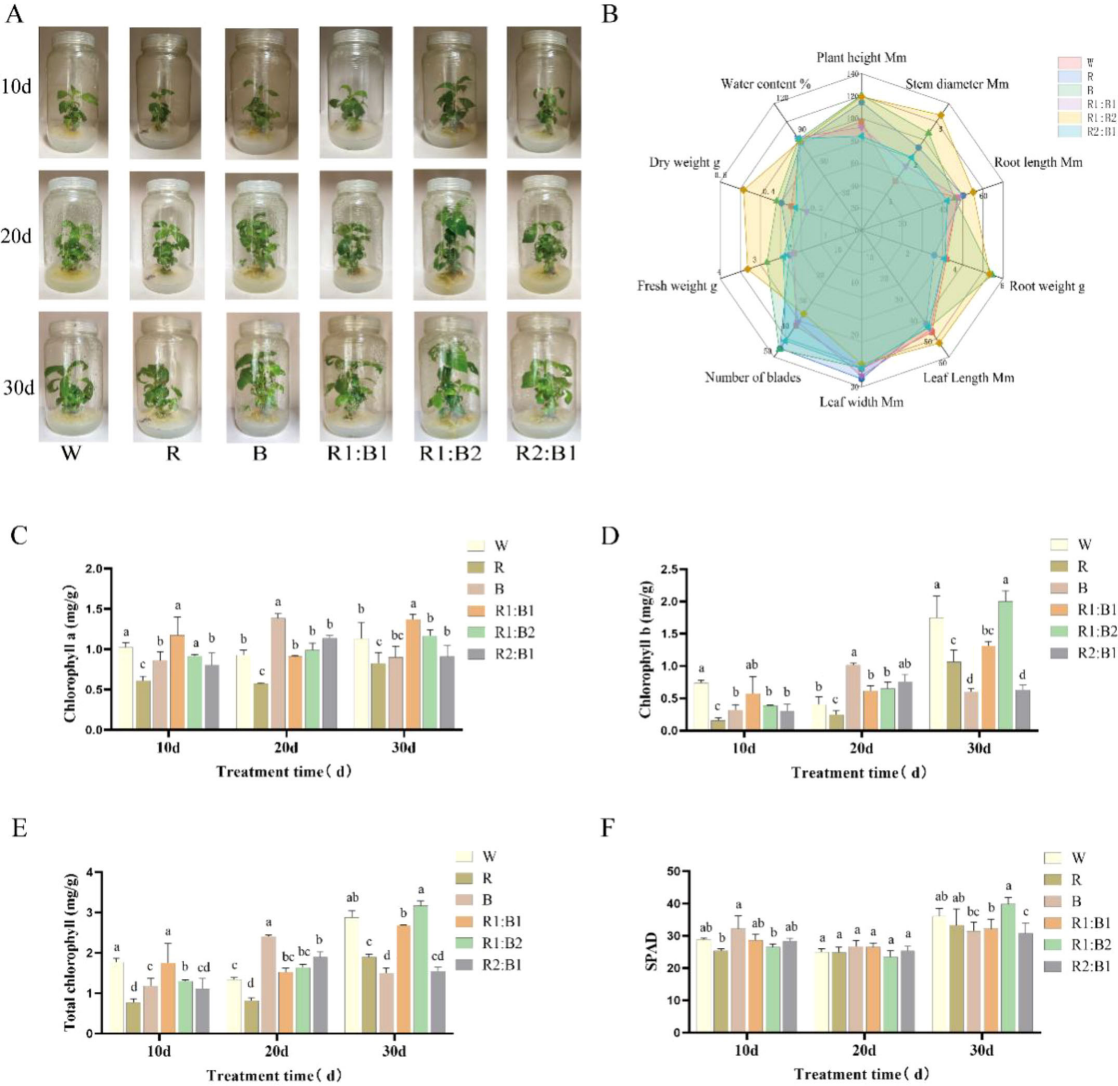
Phenotypic and physiological responses of *N. officinalis* to different light qualities. **(A)** Representative images of plant growth under various light treatments. **(B)** Radar plot of comprehensive growth indicators measured at 30 days. **(C–F)** Photosynthetic pigment contents: **(C)** chlorophyll a, **(D)** chlorophyll b, **(E)** total chlorophyll, and **(F)** SPAD values. Data are presented as mean ± SD (*n* = 3). Different lowercase letters indicate significant differences (two-way ANOVA for temporal indices, one-way ANOVA for 30-day samples, Duncan’s multiple range test, *P* < 0.05).

This study assessed the effect of light quality on the growth of *N. officinalis*, exposed to six different light treatments: white (W), monochromatic red (R), monochromatic blue (B), and three red-to-blue ratios (R1:B1, R1:B2, R2:B1). Growth was monitored over a 30-day period, with various morphological parameters, including plant height, stem diameter, root length, and both fresh and dry weight per plant. The data are presented in (**Figure 3A**). The results demonstrated that specific combinations of red and blue light significantly enhanced seedling development compared to single-spectrum treatments. The R1:B2 combination proved most effective, yielding superior outcomes for all key growth parameters. Notably, plants under R1:B2 showed the greatest increases in height, diameter, root length, and biomass, with values significantly exceeding those of the other treatments (*P* < 0.05). In contrast, seedlings under the R2:B1 and white light treatments exhibited poorer growth for multiple indicators, with differences in biomass and growth parameters being statistically significant (*P* < 0.05). These findings suggest that the R1:B2 treatment was the most favorable spectral combination for promoting seedling growth and biomass accumulation of tissue-cultured *N. officinalis* under the present controlled conditions.

### Regulation of photosynthetic pigments by light quality

To investigate the time-dependent effects of light quality on photosynthetic pigment accumulation, the levels of chlorophyll a, chlorophyll b, total chlorophyll, and SPAD values were measured in seedlings subjected to various light treatments. The light treatments included W, R, B, R1:B1, R1:B2, and R2:B1, with assessments conducted at 10, 20, and 30 days post-treatment. The results are presented in (**Figures 3C–F**). The results clearly demonstrate the time-dependent regulation of pigment accumulation by light quality, with the most significant effects observed at 20 days. At 10 days, chlorophyll a content increased by 14.48% under R1:B1 light, and SPAD values rose by 11.63% under B light, relative to the W control (*P* < 0.05). By 20 days, substantial increases in chlorophyll content were observed under the B, R1:B2, and R2:B1 treatments. Chlorophyll a content rose by 49.81%, 7.31%, and 23.01%, respectively, while chlorophyll b content surged by up to 150.16% under blue light. These differences were statistically significant (*P* < 0.05). By 30 days, the regulatory effects diminished, but R1:B2 continued to promote increases in chlorophyll content and SPAD values, with significant differences observed in chlorophyll b, total chlorophyll, and SPAD values (*P* < 0.05). These results indicate that light quality combinations differentially modulate photosynthetic pigment accumulation during specific stages of development.

### Changes in gene expression related to growth and photosynthesis

**Figure 4:**
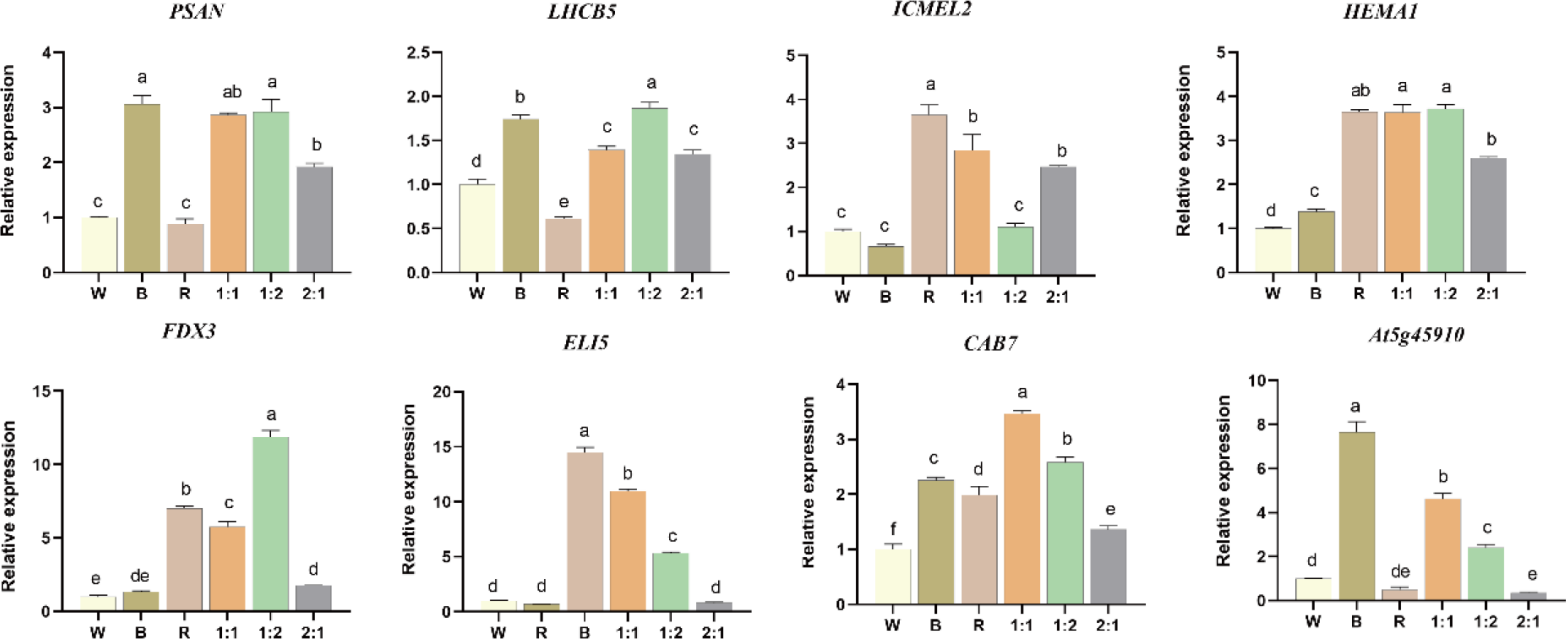
Regulation of photosynthesis-related gene expression in *N. officinalis* by light quality. Relative expression levels of *LHCB5, CAB7, PSAN, HEMA1, At5g45910, ICMEL2, ELI5*, and *FDX3* are shown. Different lowercase letters indicate significant differences (one-way ANOVA, Duncan’s multiple range test, *P* < 0.05).

To investigate how light quality modulates the expression of genes involved in photosynthesis and chlorophyll metabolism, we analyzed the relative expression levels of eight functionally distinct genes (**Figure 4**) (*LHCB5, CAB7, PSAN, HEMA1, At5g45910, ICMEL2, ELI5, FDX3*) under W, B, R, R1:B1, R1:B2, and R2:B1 light regimens via qRT-PCR. Our data demonstrate differential gene expression regulation by light quality. Expression of genes linked to the light-harvesting complex (*LHCB5* and *CAB7*) was significantly elevated under blue light or the specific R1:B2 red-blue mixture relative to other conditions. The chlorophyll biosynthesis gene *HEMA1* showed maximal expression under the R1:B2 treatment. Conversely, transcripts for the photosystem I subunit *PSAN* and certain metabolism-associated genes (eg *At5g45910, ICMEL2*) were broadly up-regulated under various mixed-light conditions. These results indicate that specific spectral combinations, particularly a R1:B2 red-to-blue ratio, can distinctly activate key genes across multiple pathways governing photosynthesis and chlorophyll metabolism.

### Determination of total phenolic acids and total flavonoids

**Figure 5:**
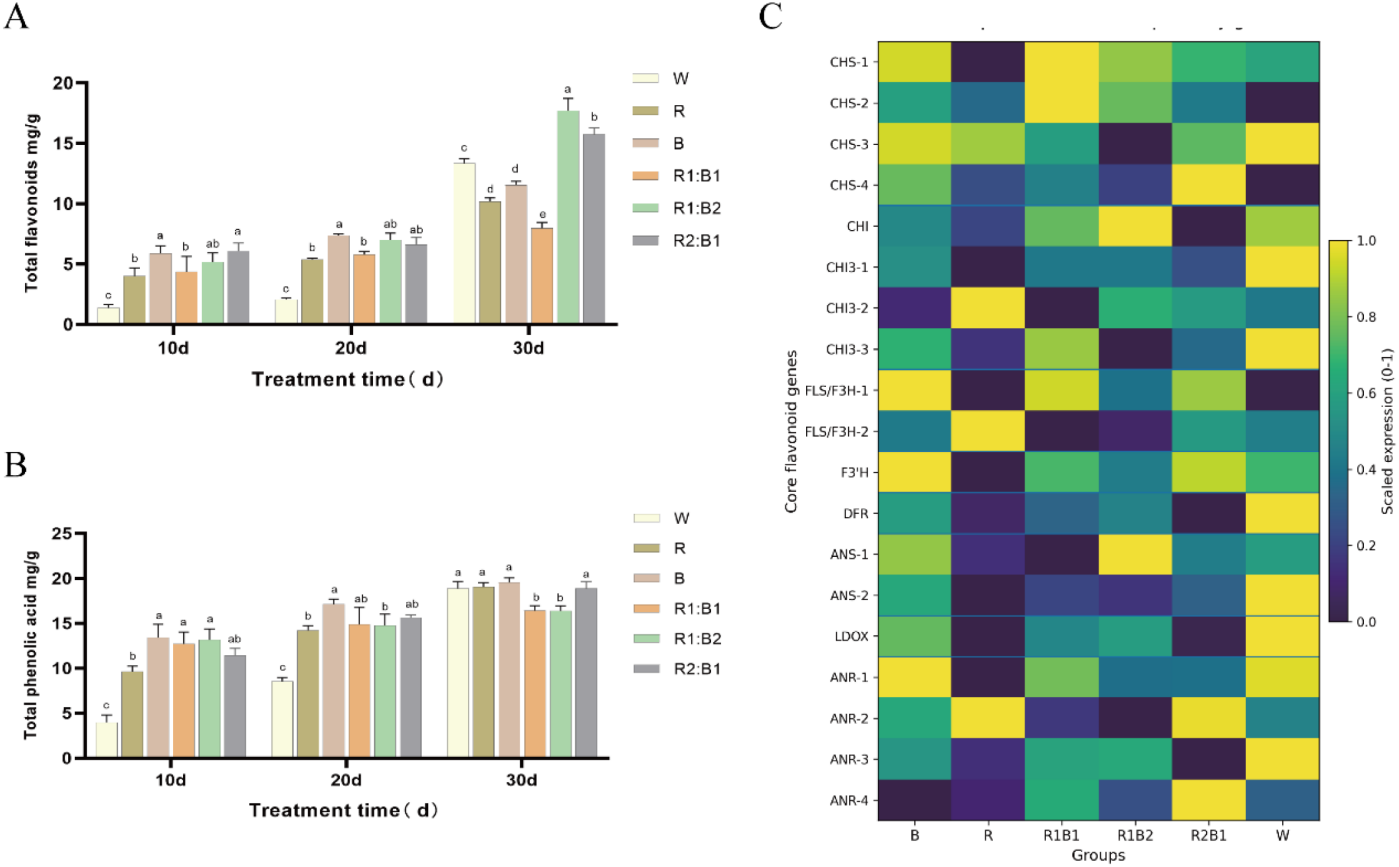
Modulation of specialized metabolism in *N. officinalis* by light quality. **(A)** Total flavonoid content; **(B)** Total phenolic acid content; **(C)** Heatmap of core flavonoid biosynthetic genes (*CHS, CHI/CHI3, FLS/F3H, F3’H, DFR, ANS, LDOX, ANR*) normalized by min–max scaling (0–1). Data in **(A, B)** are presented as mean ± SD (*n* = 3). Different lowercase letters denote significant differences (two-way ANOVA, Duncan’s multiple range test, *P* < 0.05).

To evaluate the effects of light quality and red-blue ratios on leaf secondary metabolite accumulation, total flavonoids and total phenolic acids were measured at 10, 20, and 30 d under W, R, B, R1:B1, R1:B2, and R2:B1 treatments. Total flavonoid content increased progressively with treatment duration (**Figure 5A**). At 10 d, the W treatment showed the lowest flavonoid level, whereas B and R2:B1 were relatively higher. At 20 d, B remained among the higher treatments. By 30 d, flavonoid content increased markedly under R1:B2 and reached the highest level among all treatments, whereas W and R2:B1 were intermediate and R1:B1 remained the lowest. Total phenolic acid content also increased over time, but its response pattern differed from that of total flavonoids (**Figure 5B**). At 10 d, B, R1:B1, and R1:B2 showed higher levels than W, and B reached the highest level at 20 d. At 30 d, W, R, B, and R2:B1 maintained similarly high phenolic acid contents, whereas R1:B1 and R1:B2 were comparatively lower. These results indicate that secondary metabolite accumulation in *N. officinalis* was regulated by light quality in a growth-stage-dependent manner, and that total flavonoids and total phenolic acids exhibited distinct spectral response patterns.

Because transcriptome sequencing was conducted only at 30 d, gene-expression analysis was used to interpret treatment differences at the late stage rather than the temporal trends observed at 10 and 20 d. Expression profiling of core flavonoid biosynthetic genes revealed clear treatment-specific and gene-specific patterns (**Figure 5C**). Structural genes associated with flavonoid biosynthesis, including *CHS, CHI, FLS/F3H, F3’H, DFR, ANS, LDOX*, and *ANR*, showed heterogeneous responses among treatments, and no single light regime induced uniformly high expression of all genes. Although several genes or isoforms displayed relatively higher expression under R1:B2 and R2:B1, others showed higher expression under W, B, or R1:B1. Thus, the elevated flavonoid content under R1:B2 at 30 d did not coincide with the highest expression of all core flavonoid biosynthetic genes. Instead, the transcriptome data suggest that treatment-dependent flavonoid accumulation at 30 d was associated with coordinated transcriptional shifts in multiple pathway genes rather than a uniform expression response.

### Changes in gene expression related to alkaloid metabolism

**Figure 6:**
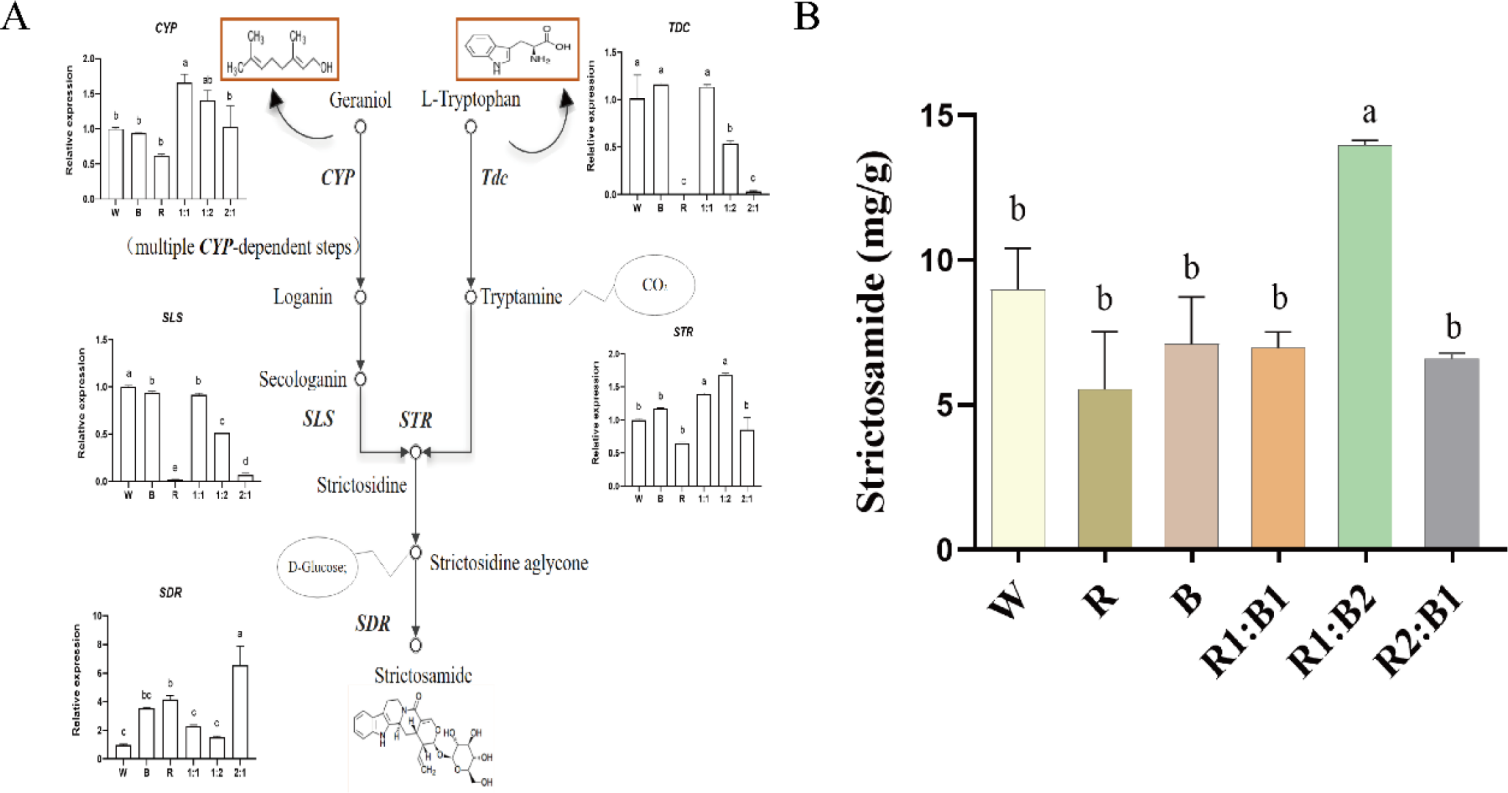
Effects of light quality on strictosamide biosynthesis in *N. officinalis.* **(A)** Schematic of the strictosamide biosynthetic pathway and qRT-PCR quantification of key biosynthetic genes (*CYP, TDC, SLS, STR, SDR*). **(B)** High-performance liquid chromatography (HPLC) quantification of strictosamide content. Data are presented as mean ± SD (*n* = 3). Different lowercase letters indicate significant differences (one-way ANOVA, Duncan’s multiple range test, *P* < 0.05).

To determine how the six treatments (W, B, R, R1:B1, R1:B2, and R2:B1) affect strictosamide (isovincosamide lactam) production and the expression of associated biosynthetic genes, we measured strictosamide content (**Figure 6B**) and quantified the transcript levels of key biosynthetic genes by qRT-PCR (**Figure 6A**). Strictosamide accumulation was maximized under the R1:B2 treatment, which was significantly higher than all other groups, whereas W, B, R, R1:B1, and R2:B1 were statistically indistinguishable (**Figure 6B**). Gene expression patterns differed by enzymatic step: *CYP* was highest in R1:B1 and remained elevated in R1:B2; *TDC* was maintained at high levels in W, B, and R1:B1 but was reduced in R1:B2 and minimal in R and R2:B1; *SLS* showed the strongest expression in W, moderate expression in B and R1:B1, pronounced repression in R and R1:B2, and the lowest level in R2:B1; *STR* was selectively induced in R1:B1 and R1:B2; and *SDR* was most strongly induced in R2:B1 (**Figure 6A**). Collectively, these results suggest that R1:B2 is the most favorable condition for strictosamide accumulation, while transcriptional regulation along the pathway is treatment-dependent and enzyme-specific (**Figures 6A, B**). This result implies that strictosamide accumulation under R1:B2 is likely governed by coordinated pathway regulation rather than the maximal induction of any single biosynthetic gene.

## Discussion

For medicinal plants, changes in specialized metabolites are of particular importance because they are directly related to pharmacological value. Therefore, in the present study, total flavonoids and strictosamide were treated as key metabolic endpoints for evaluating the biological significance of light-quality regulation in *N. officinalis*. Although the present study was conducted using established physiological and molecular approaches, its value lies in integrating seedling growth, photosynthetic traits, active metabolite accumulation, and related transcriptional responses in *N. officinalis.*

Light quality is a key environmental signal regulating plant development and secondary metabolism, including phenolics, terpenoids, and alkaloids (Trivellini et al., 2023). Consistent with the principle that specific photoreceptors activate distinct metabolic pathways, our data show that the R1:B2 red-blue ratio most strongly upregulated transcripts for light-harvesting proteins *LHCB5* and *CAB7* and chlorophyll biosynthesis *HEMA1*, indicating enhanced photochemical capacity. While blue light alone transiently boosted chlorophyll content by 20 days, its effect diminished by 30 days, and pure red light broadly suppressed gene expression 1,458 genes down-regulated. This aligns with general observations that blue light promotes photosynthetic pigments whereas red light can restrict biomass and pigment accumulation in some species (Song et al., 2022; Naznin et al., 2019a; Naznin et al., 2019b). Critically, however, *N. officinalis* requires a blue-dominant spectrum to sustain high chlorophyll and growth, distinguishing it from species that thrive under red-dominant conditions.

Although strictosamide accumulation was highest under the R1:B2 treatment, this did not coincide with the highest expression of all examined biosynthetic genes, and such a pattern is not necessarily contradictory. MIA biosynthesis is a multistep and highly coordinated network in which final metabolite abundance depends not only on the transcript levels of individual genes, but also on precursor availability, pathway flux partitioning, branch-point regulation, downstream conversion, compartmentation, and metabolite turnover (Pan et al., 2016; Verma et al., 2012). In the present study, transcriptional responses were clearly step-specific rather than uniform: *CYP* remained relatively high under R1:B1 and R1:B2, *STR* responded preferentially to mixed red-blue treatments, whereas *TDC, SLS*, and *SDR* showed distinct treatment preferences. These results suggest that the superior strictosamide accumulation under R1:B2 was more likely the consequence of coordinated regulation across multiple biosynthetic steps than of maximal induction of all upstream genes. This interpretation is also consistent with the broader effects of light quality on secondary metabolism (Wang et al., 2022). Blue light is widely recognized as an important signal for flavonoid biosynthesis, whereas red light is often associated with terpenoid-related pathways (Zhang S. et al., 2021; Sun et al., 2025). However, the optimal spectral ratio is strongly species dependent: for example, phenolic acid accumulation in Salvia miltiorrhiza, biomass formation in Centella asiatica (Zhang et al., 2020; Song et al., 2022). Thus, the favorable effect of R1:B2 on strictosamide accumulation in *N. officinalis* likely reflects a species-specific integration of light signaling and metabolic regulation rather than a simple one-to-one response of individual genes. From a signaling perspective, this response may be associated with the balance between blue- and red-light pathways. HY5 is considered a major positive regulator downstream of multiple photoreceptors, whereas PIF-family factors mediate phytochrome-dependent responses and can reshape both photosynthetic gene expression and specialized metabolism (Jing and Lin, 2020; Li et al., 2026). Although HY5/PIF regulation was not directly examined in this study, a blue-enriched red-blue regime may better coordinate photosynthetic competence with MIA-related metabolism than monochromatic light alone. In addition, strictosamide metabolism in Nauclea-type alkaloid systems is not static; strictosamide has been reported as a central alkaloid scaffold that can be further converted into downstream derivatives, including vincoside lactam-type compounds, and strictosamide-to-vincoside lactam conversion has been demonstrated under acidic conditions (Songoen et al., 2022). Therefore, the strictosamide content detected at a given time point may reflect not only biosynthetic input, but also subsequent structural conversion and metabolic fate, which may further explain why metabolite accumulation does not always show a simple one-to-one correspondence with the expression pattern of upstream biosynthetic genes.

Light-regulated flavonoid accumulation is widely reported, particularly under blue or blue-enriched spectra. In the present study, total flavonoid accumulation in *N. officinalis* also showed a clear response to spectral composition, but this response was stage dependent, with the most pronounced increase occurring under the R1:B2 treatment at 30 d. Because transcriptome sequencing was performed only at 30 d, the corresponding gene-expression data are more suitable for interpreting late-stage treatment differences than for explaining the dynamic changes observed at 10 and 20 d. In *Epimedium sagittatum*, blue light promoted the accumulation of multiple flavonoids, and in *Scutellaria baicalensis*, blue-dominant treatments enhanced both biomass and flavonoid content. In contrast, *Anoectochilus roxburghii* showed the highest total flavonoid level under a combined red-blue treatment rather than under monochromatic light, indicating that flavonoid responses to spectral quality are species-specific and do not follow a single universal pattern (Yang et al., 2022; Zhang et al., 2022; Gam et al., 2020). Therefore, the flavonoid results in the present study should not be treated as incidental. Instead, they suggest that light quality in *N. officinalis* simultaneously influenced at least two metabolically important branches, namely the strictosamide-related alkaloid pathway and the flavonoid pathway. However, the elevated total flavonoid content under R1:B2 did not coincide with uniformly higher expression of all core flavonoid biosynthetic genes, indicating that flavonoid accumulation was not determined by the maximal expression of every structural gene at a single sampling point. These transcriptomic results suggest that flavonoid accumulation was associated with coordinated expression changes in representative flavonoid-biosynthesis genes under different light treatments.

This partial inconsistency between flavonoid content and transcript abundance is not unusual in plant secondary metabolism. In Arabidopsis, UV-B exposure largely abolished the correlations between flavonoid-biosynthetic transcripts and flavonoid levels, indicating that transcript abundance and metabolite accumulation can become uncoupled under specific environmental conditions (Schulz et al., 2021). A similar phenomenon was reported in Ginkgo biloba, where both UV-B and NaCl treatments promoted flavonoid accumulation, but through distinct transcriptional responses, and the combined treatment did not further increase flavonoid content despite comparable expression patterns of many structural genes (Ni et al., 2017). In breadfruit scions, elevated total flavonoid content was associated mainly with the up-regulation of *CHS*, whereas not all downstream structural genes showed corresponding increases, suggesting that higher flavonoid accumulation does not require synchronous up-regulation of the entire pathway (Zhou and Underhill, 2023). Likewise, in Sophora flavescens, the transcript levels of *PAL* and *C4H* (cinnamate 4-hydroxylase) were reported not to correlate directly with flavonoid content, further supporting the view that upstream gene expression alone is insufficient to predict final metabolite abundance (Lee et al., 2018). Taken together, these findings suggest that the higher total flavonoid content observed under R1:B2 in the present study is more reasonably interpreted as the integrated outcome of coordinated regulation across multiple pathway steps, rather than the maximal expression of all flavonoid-biosynthetic genes at a single sampling point. This also suggests that the favorable effect of the R1:B2 treatment was not restricted to a single metabolite class, but may reflect a broader reprogramming of secondary metabolism under a blue-enriched red-blue spectrum.

Despite these promising results, several limitations should be acknowledged. The present study was limited to tissue-cultured seedlings grown under short-term controlled conditions, and the findings therefore mainly reflect seedling-stage responses to different light spectra. Whether the R1:B2 ratio can consistently maintain higher biomass accumulation and metabolite production over longer cultivation periods and across different developmental stages still requires further investigation. In addition, because the experiments were conducted under controlled environmental conditions, caution is needed when applying these results directly to the open-field cultivation of woody medicinal plants. Even so, the present findings may provide a useful reference for light management during the seedling stage and support further research on controlled cultivation and acclimatization.

**Figure 7:**
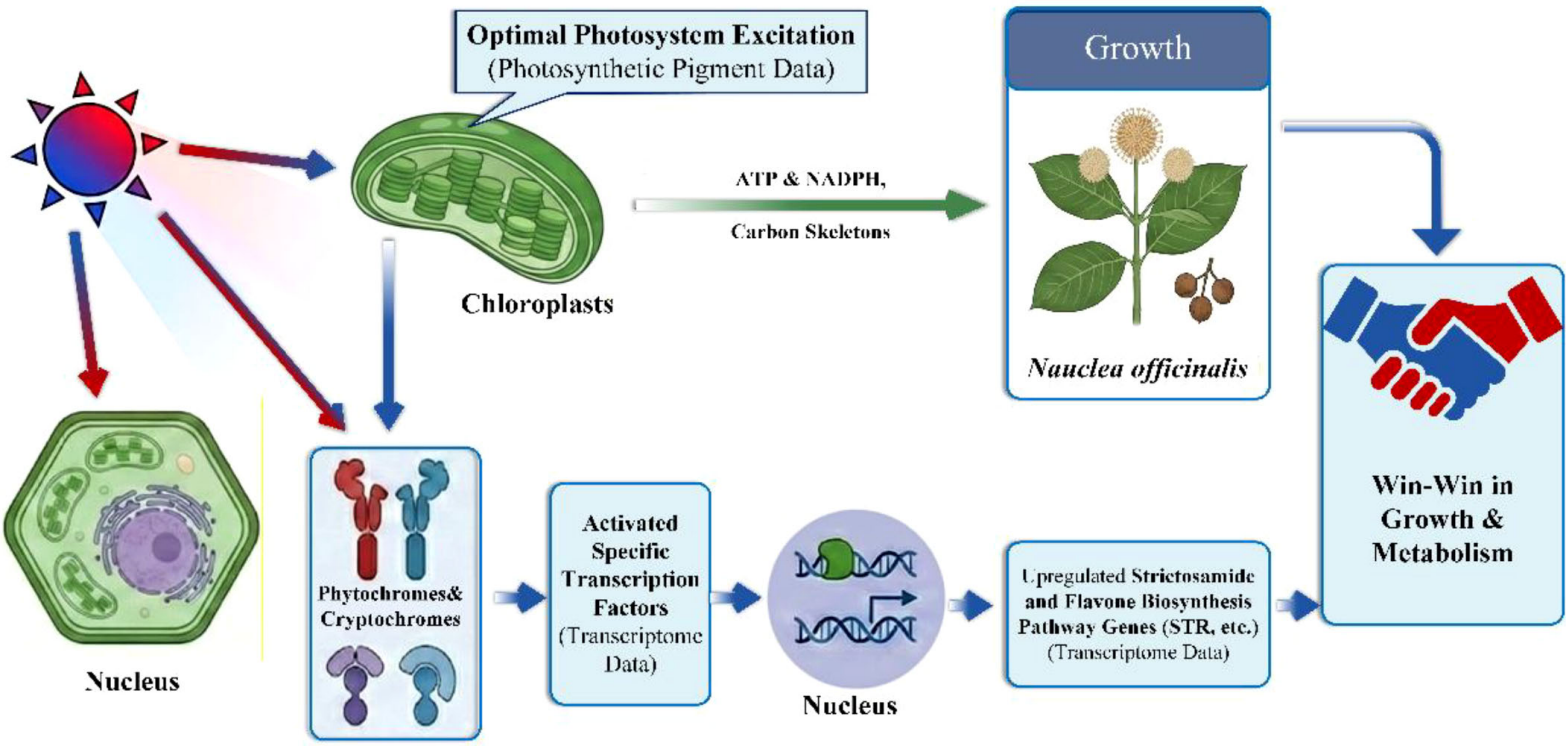
Model of light-regulated growth and specialized metabolism in *N. officinalis*. This schematic illustrates the coordination between primary metabolism, photosynthetic processes, and strictosamide biosynthesis under different light treatments. Chloroplast-derived energy and carbon precursors support biomass accumulation, while light-responsive photoreceptors and downstream regulatory factors modulate the biosynthesis of specialized metabolites. This model summarizes the key trends observed in the present study and does not represent a fully elucidated regulatory mechanism.

Future work should integrate metabolomic analyses, enzyme assays, and hormone measurements to clarify how coordinated changes in *CYP, TDC, SLS, STR*, and *SDR* contribute to strictosamide accumulation under different light qualities. Testing dynamic light regimes that adjust red−blue proportions across developmental stages may better match changing metabolic demands. The incorporation of green and far−red wavelengths may refine photosynthetic efficiency and photomorphogenesis. could reveal conserved and divergent light responses. Finally, elucidating the photoreceptor signalling networks that underlie the unique blue−dominant requirement of *N. officinalis* will guide the rational design of LED spectra for optimizing growth and specialized metabolism in medicinal plants (**Figure 7**).

## Conclusions

In conclusion, different light qualities distinctly affected the growth, photosynthetic traits, and specialized metabolism of tissue-cultured *N. officinalis* seedlings under controlled conditions. Among the tested treatments, the blue-enriched red-blue ratio R1:B2 was the most favorable for coordinating seedling growth, chlorophyll accumulation, and strictosamide-related metabolism. This treatment also enhanced total flavonoid accumulation at 30 d, suggesting that its regulatory effect was not restricted to a single metabolite class. Transcriptomic and qRT-PCR analyses further suggested that this response was associated with coordinated regulation of photosynthesis-related genes and specialized metabolic pathways. In contrast, monochromatic red light showed a generally suppressive effect on transcriptional activity, whereas mixed red-blue spectra better supported integrated physiological regulation. These findings indicate that spectral composition is an important factor shaping seedling-stage performance of *N. officinalis* and provide a reference for nursery-stage light regulation and future studies on controlled cultivation and metabolic quality formation.

## Data Availability

The datasets presented in this study can be found in online repositories. The names of the repository/repositories and accession number(s) can be found below: https://www.ncbi.nlm.nih.gov/, PRJNA1419266.
